# Correction to: Urinary extracellular vesicle‑derived miR‑126‑3p predicts lymph node invasion in patients with high‑risk prostate cancer

**DOI:** 10.1007/s12032-025-02636-1

**Published:** 2025-03-21

**Authors:** Liang Dong, Cong Hu, Zehua Ma, Yiyao Huang, Greg Shelley, Morgan D. Kuczler, Chi‑Ju Kim, Kenneth W. Witwer, Evan T. Keller, Sarah R. Amend, Wei Xue, Kenneth J. Pienta

**Affiliations:** 1 Department of Urology, Ren Ji Hospital, Shanghai Jiao Tong University School of Medicine, Shanghai, 200127 China; 2 The Brady Urological Institute, Johns Hopkins University School of Medicine, 600 North Wolfe Street, Baltimore, MD 21287 USA; 3 Department of Urology, Guizhou Provincial People’s Hospital, Guiyang, 550001 China; 4 Department of Laboratory Medicine & Guangdong Engineering and Technology Research Center for Rapid Diagnostic Biosensors, Nanfang Hospital Southern Medical University, Guangzhou, 510515 China; 5 Department of Molecular and Comparative Pathobiology, Johns Hopkins University School of Medicine, Baltimore, MD 21205 USA; 6 Biointerfaces Institute, University of Michigan, Ann Arbor, MI 48109 USA; 7 Department of Urology, University of Michigan, Ann Arbor, MI 48109 USA; 8 Department of Urology, Ren Ji Hospital, Shanghai Jiao Tong University School of Medicine, 160 Pujian Road, Pudong New Area, Shanghai, 200127 China

**Correction to: Medical Oncology (2024) 41:169** 10.1007/s12032-024-02400-x

The original version of this article unfortunately contained a mistake in Fig. 2.

In Fig. 2 of this article, two errors were originally present:

(a) In Fig. 2B, the particle size distribution plots displayed incorrect data; the correct distribution plots have now been used.

(b) In Fig. 2C, the CD81 positive/negative control bands displayed incorrect data; the correct band has now been used.

Incorrect version
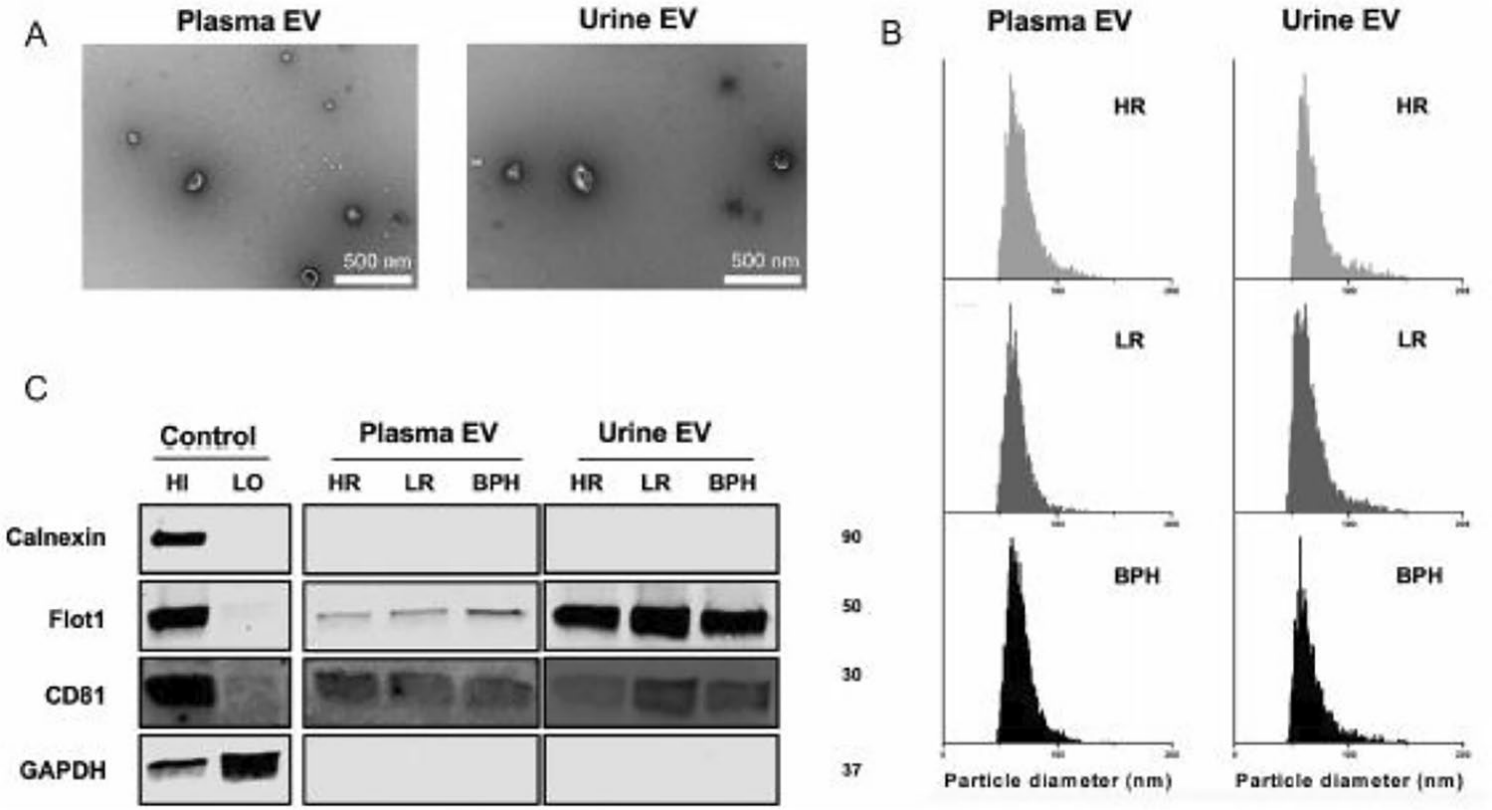


Correct version (Fig. 2)

**Fig. 2:**
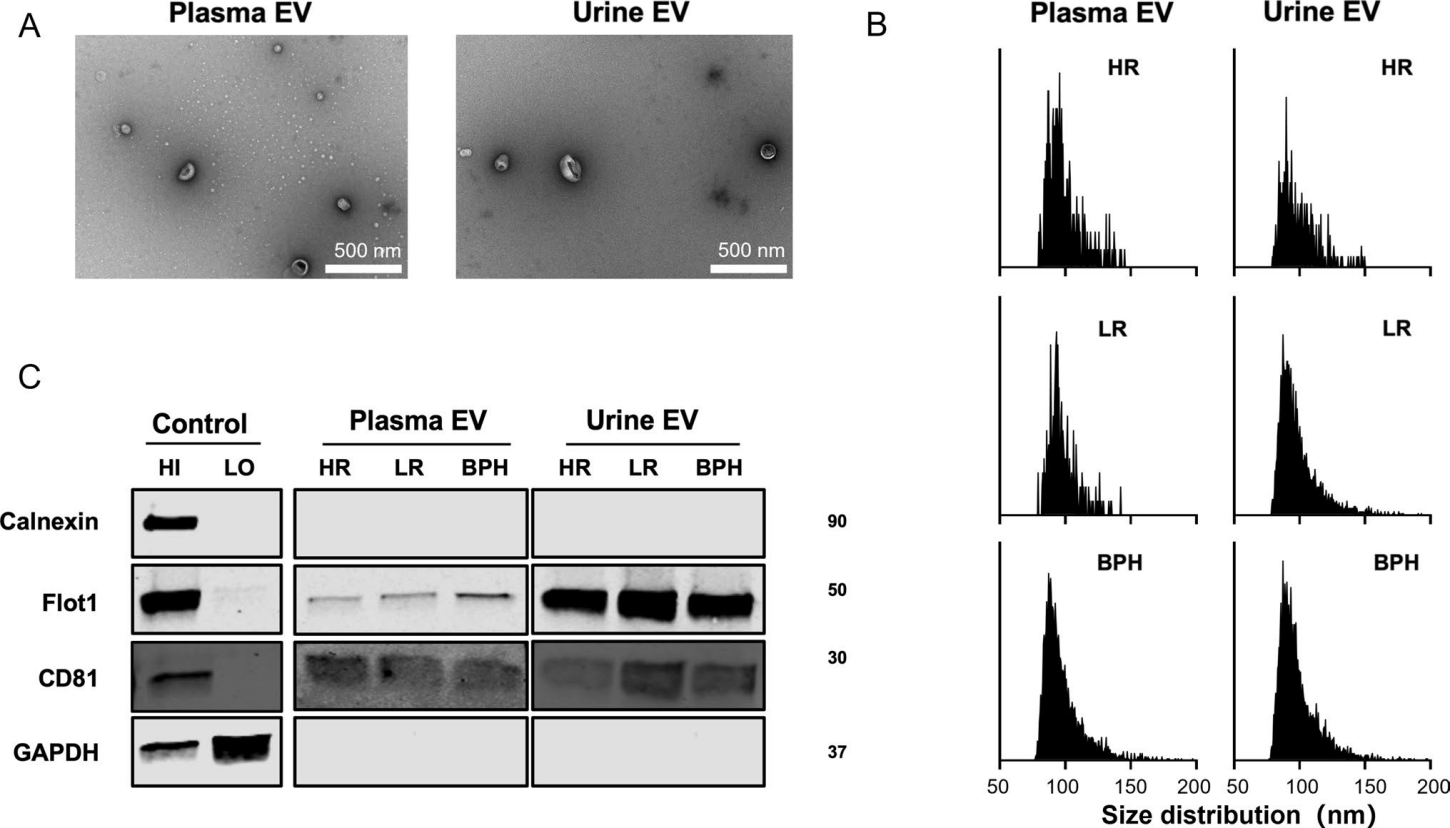
Characterization of separated EVs from plasma and urine. **A** TEM confirms the cup-shaped EVs. Scale bar is 500 nm. **B** The size distribution and concentrations of separated EVs is analyzed via nFCM. **C** EV markers are verified using western blotting. Abbreviations: *HI* high abundance control, *LO* low abundance control, *BPH* benign prostate hyperplasia, *HR* high risk, *LR* low-risk

The original article has been corrected.

